# Transformer-Based Detection and Clinical Evaluation System for Torsional Nystagmus

**DOI:** 10.3390/s25134039

**Published:** 2025-06-28

**Authors:** Ju-Hyuck Han, Yong-Suk Kim, Jong Bin Lee, Hantai Kim, Jong-Yeup Kim, Yongseok Cho

**Affiliations:** 1 Department of Artificial Intelligence, Konyang University, Daejeon 32992, Republic of Korea; jhhan@konyang.ac.kr (J.-H.H.); yongsuk@konyang.ac.kr (Y.-S.K.); 2 Department of Otolaryngology, Konyang University Hospital, Daejeon 32992, Republic of Korea; rogue25@kyuh.ac.kr (J.B.L.); chriskht@kyuh.ac.kr (H.K.); 3 Department of Medical IT Engineering, Konyang University, Daejeon 32992, Republic of Korea

**Keywords:** torsional nystagmus, 1D-CNN, nystagmus detection, benign paroxysmal positional vertigo, torsion transformer

## Abstract

**Motivation:** Benign paroxysmal positional vertigo (BPPV) is characterized by torsional nystagmus induced by changes in head position, where accurate quantitative assessment of subtle torsional eye movements is essential for precise diagnosis. Conventional videonystagmography (VNG) techniques face challenges in accurately capturing the rotational components of pupil movements, and existing automated methods typically exhibit limited performance in identifying torsional nystagmus. **Methodology:** The objective of this study was to develop an automated system capable of accurately and quantitatively detecting torsional nystagmus. We introduce the Torsion Transformer model, designed to directly estimate torsion angles from iris images. This model employs a self-supervised learning framework comprising two main components: a Decoder module, which learns rotational transformations from image data, and a Finder module, which subsequently estimates the torsion angle. The resulting torsion angle data, represented as time-series, are then analyzed using a 1-dimensional convolutional neural network (1D-CNN) classifier to detect the presence of nystagmus. The performance of the proposed method was evaluated using video recordings from 127 patients diagnosed with BPPV. **Findings:** Our Torsion Transformer model demonstrated robust performance, achieving a sensitivity of 89.99%, specificity of 86.36%, an F1-score of 88.82%, and an area under the receiver operating characteristic curve (AUROC) of 87.93%. These results indicate that the proposed model effectively quantifies torsional nystagmus, with performance levels comparable to established methods for detecting horizontal and vertical nystagmus. Thus, the Torsion Transformer shows considerable promise as a clinical decision support tool in the diagnosis of BPPV. **Key Findings:** Technical performance improvement in torsional nystagmus detection; System to support clinical decision-making for healthcare professionals.

## 1. Introduction

Nystagmus is defined as an involuntary, rhythmic oscillation of the eyes and is an essential clinical indicator for diagnosing peripheral or central vestibular disorders [1,2]. Benign paroxysmal positional vertigo (BPPV), the most prevalent vestibular disorder, manifests as brief episodes of dizziness triggered by positional changes, accompanied by transient nystagmus [3,4,5]. Specifically, posterior canal BPPV exhibits torsional nystagmus with an upward-beating torsional movement, enabling clinicians to identify both the affected ear and the involved semicircular canal [6,7,8]. Traditionally, clinicians diagnose nystagmus by visually interpreting eye movements recorded via videonystagmography (VNG), focusing on direction and velocity [9,10,11]. However, subtle rotational components are challenging to detect visually, particularly for less experienced practitioners, leading to reduced diagnostic accuracy [12,13,14]. Even experienced specialists may encounter difficulties, resulting in misdiagnosis, unnecessary additional testing, and variability in interpretation between examiners [15,16].

Recent advancements in automated nystagmus detection have leveraged computer vision and deep learning methodologies. Horizontal and vertical nystagmus detection through pupil tracking methods has achieved accuracy exceeding 90%. For instance, Lim et al. (2019) developed a deep learning-based diagnostic support system trained on 1005 nystagmus video samples, achieving an F1-score of 0.89 for classifying horizontal and vertical nystagmus [9]. Mun et al. (2024) implemented real-time horizontal nystagmus detection using video-oculography data and a pupil-tracking model based on a 1-dimensional convolutional neural network (1D-CNN), obtaining high sensitivity (94.06%) and specificity (86.39%) [16].

Despite these advancements, the automated detection of torsional nystagmus remains problematic. Zhang et al. (2021) utilized optical flow-based features combined with the TBSIN model for analyzing torsional nystagmus in 77 posterior canal BPPV patients, achieving an F1-score of approximately 81% [17], significantly lower than the accuracy of approximately 90% for horizontal and vertical nystagmus detection using the same model. This performance gap stems primarily from the inherent difficulties in accurately measuring torsional eye movements. Li and Yang (2023) aimed to detect vertical and torsional nystagmus using over 10,000 video segments, achieving roughly 92% accuracy primarily for vertical components but did not quantitatively evaluate torsional movements [6]. Furthermore, existing deep learning methods typically classify nystagmus as either present or absent without quantifying torsional angles, thus limiting their clinical interpretability. Pham et al. (2022) proposed a hybrid model combining image classification and signal processing, demonstrating diagnostic potential for BPPV, yet similarly lacking quantitative assessment of torsional components [18].

Therefore, this study aims to develop a novel deep learning model for accurately detecting torsional nystagmus and quantitatively assessing torsion angles, providing clinicians with interpretable diagnostic metrics. Inspired by the iris pattern analysis method introduced by Ong and Haslwanter (2010) [19], we propose the Torsion Transformer model, which integrates continuous torsion angle estimation and anomaly detection capabilities. We evaluate the proposed model’s performance using publicly available datasets and clinical patient recordings, seeking to address existing limitations through comparative analysis with previously established methods.

## 2. Materials and Methods

### 2.1. Datasets

The dataset for this study consists of two sources. The first is the CASIA-Iris-Degradation dataset, containing 36,539 infrared images from 255 subjects and corresponding iris and pupil parameters [20,21]. Ground-truth annotations were established by pairing 32,962 images with their respective parameters. The second dataset includes retrospective VNG recordings from 750 BPPV patients collected at Konyang University Hospital between 2013 and 2023 (IRB No. KYUH 24-06-025). Data anonymization involved pseudonymization, removal of identifiable information, and data categorization. Inclusion criteria covered adults aged 19–80 years diagnosed with BPPV and who underwent VNG. Exclusion criteria encompassed refractory BPPV cases, other peripheral positional vertigo types, other ear disorders, and central nervous system disorders. Clinical experts annotated nystagmus events temporally.

To summarize, both the CASIA-Iris-Degradation dataset and the dataset collected at Konyang University Hospital comprise VNG recordings from patients diagnosed with BPPV. However, the primary reason for not relying solely on the publicly available dataset is its limited representation of posterior canal BPPV—the specific subtype associated with torsional nystagmus, which is the focus of this study. In the CASIA-Iris-Degradation dataset, only two subjects exhibit posterior canal involvement, corresponding to merely two relevant VNG recordings. To address this limitation, we retrospectively collected additional data from Konyang University Hospital. When extracting only the VNG recordings corresponding to torsional nystagmus, the CASIA dataset includes 2 out of 255 subjects, whereas the Konyang dataset provides 125 out of 750 patients. To ensure consistency, both datasets were standardized to a resolution of 320 × 120 pixels, yielding a total video duration of 12,891 s. Each video was recorded at 30 frames per second and subsequently segmented accordingly. The segmented frames were cropped to 180 × 120 pixels to isolate the left and right eyes, resulting in a total of 421,662 images. For optimal performance and accurate torsion estimation, the combined dataset was divided into training (295,164 frames, 70%), validation (84,333 frames, 20%), and test (42,165 frames, 10%) sets.

### 2.2. Preprocessing

**Figure 1:**
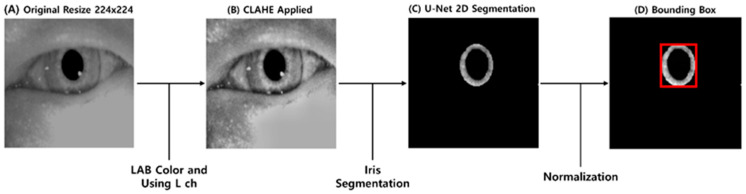
Overview of the preprocessing workflow and resulting outputs. (**A**) shows a resized VNG frame (from 180 × 120 pixels to 256 × 256 pixels). (**B**) displays the result of applying CLAHE to the L-channel in the LAB color space. (**C**) presents the iris segmented using a U-Net-based method. (**D**) illustrates the bounding box generated on the normalized segmented iris.

In this study, torsional nystagmus detection is performed through iris segmentation from VNG images. This method addresses the challenge of extracting rotational information from elliptical objects, such as the pupil, by using iris patterns to estimate torsional angles. Figure 1 shows the preprocessing workflow and its results fragmentarily. The preprocessing workflow begins with (A) resizing left and right VNG images separately to 224 × 224 pixels to ensure compatibility with the iris segmentation model. Noise elements in the raw ocular images, such as reflections and eyelids, are mitigated by (B) converting the images to the LAB color space and applying Contrast-Limited Adaptive Histogram Equalization (CLAHE) to the luminance channel, thereby enhancing the boundary clarity between the iris and sclera. Subsequently, a (C) pretrained U-Net model is utilized for pixel-wise iris segmentation. This model is a modified version of a publicly available pretrained network on HuggingFace, adapted for iris segmentation tasks. The modified model adopts ES-Net, which has recently demonstrated strong performance in iris segmentation tasks by incorporating an attention mechanism and the Efficient Spatial Pyramid Block (ESP block) for improved segmentation accuracy and computational efficiency [22,23]. To accommodate the input requirements of this model, the image resolution is adjusted to 224 × 224. Detailed architectural specifications can be found in Pourafkham et al. [23]. In the next step, the segmented iris regions are enclosed using (D) bounding boxes defined by their minimum enclosing circles and normalized to 64 pixels in diameter. Data augmentation involves randomly rotating the iris images between −15° and +15°, producing paired original–rotated inputs. These pairs are used to train the Decoder module of the Torsion Transformer, with the corresponding rotation angles serving as labels for the Finder module.

### 2.3. Torsion Transformer

**Figure 2:**
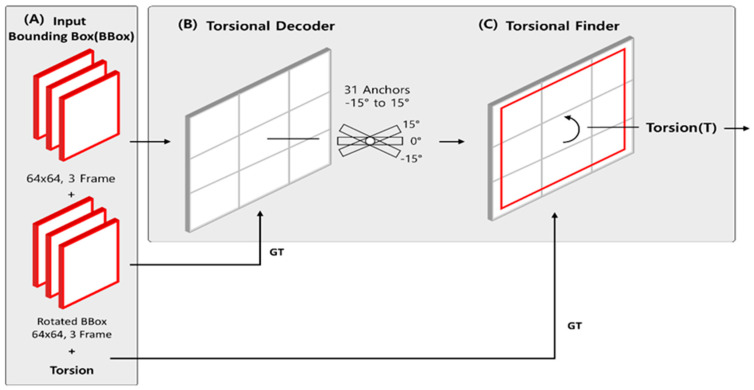
Overall architecture of the proposed Torsion Transformer. (**A**) Illustrates the input configuration to the Torsion Transformer, which includes a preprocessed iris image (Bounding Box), its rotated version at a given angle, and the corresponding rotation angle as a label. The architecture consists of two core modules: the (**B**) Decoder and the (**C**) Finder. The Decoder receives the original (unrotated) iris image and aims to synthesize its rotated counterpart. In this process, 31 anchor tensors (including the original image) corresponding to rotation angles from −15° to +15° are learned during training. The Finder, shown in (**C**), is designed to perform regression to estimate the torsional angle at which the iris image is rotated over a 3-frame segment. This module leverages the anchor tensors learned by the Decoder to facilitate angle prediction.

The Torsion Transformer is a neural network architecture specifically developed to directly predict the torsional angle of the iris from ocular images. Unlike conventional Transformer-based models that adopt an encoder–decoder structure, the proposed model operates in reverse: it first applies a rotational transformation to the input image via a Decoder and subsequently estimates the rotation angle using a Finder module. The term Torsion Transformer reflects the model’s reversed architectural workflow and its explicit prediction of torsional angle, distinguishing it from standard Transformer architectures. Figure 2 illustrates the architecture of the Torsion Transformer. In (A), the input consists of a preprocessed image (Bounding Box), a target rotation angle, and the image rotated by that angle. The original image is resized to 64 × 64 pixels and provided as a three-frame sequence. The rotated image serves as the ground truth (GT) for the Decoder module in (B), while the associated rotation angle is used as the GT for the Finder module in (C).

The model comprises two primary modules: the (B) Torsion Decoder and the (C) Torsion Finder, both of which process the same iris bounding box input in parallel. First, the Torsion Decoder (B) generates 31 anchor images by rotating the original image from −15° to +15° in 1° increments. This anchor-based learning strategy explicitly encodes rotational states as feature representations, effectively capturing variations in iris patterns caused by torsion. These intermediate anchor features are essential for enabling precise torsional angle estimation. The output of the Decoder is compared with the actual rotated image for supervision. Subsequently, the Torsion Finder (C) utilizes the anchor features generated by the Decoder to estimate the torsional angle. In this regression stage, the original image is input, and the predicted torsional angle is compared against the ground truth.

**Figure 3:**
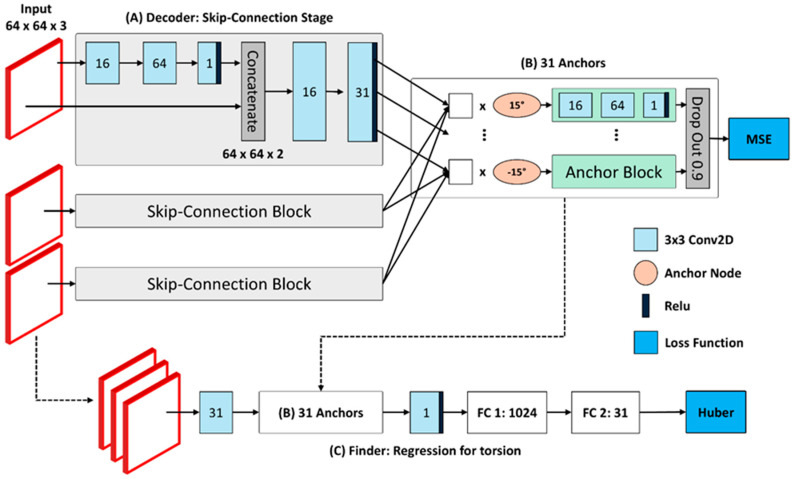
Presents the detailed architecture of the Torsion Transformer. Panel (**A**) depicts the preceding Decoder structure within the Transformer, which is primarily characterized by its use of skip connections—an essential mechanism in the design. Panel (**B**) illustrates the operational framework of the 31 anchors previously introduced at the conceptual level, demonstrating how they function in practice. Panel (**C**) corresponds to the Finder module of the Transformer, which performs regression to estimate the torsional angle. In the diagram, light blue blocks represent 3 × 3 two-dimensional convolutional layers, while orange circles denote nodes that compute key weights corresponding to each anchor angle. The activation function ReLU, which plays a crucial role in non-linearity, is indicated in dark blue. The loss functions associated with each module are marked in blue boxes.

In summary, the input data fed into the Transformer consists of iris bounding box images, from which most noise unrelated to the iris pattern has been removed through preprocessing. The Decoder is trained to generate images that resemble the rotated versions of the original image at specific angles, while the Finder estimates the torsional angle based solely on the original image. During this process, the Decoder and its anchors learn discriminative features representing the transformation between original and rotated images. In this way, the Torsion Transformer is capable of learning fine-grained rotational features from limited input data (i.e., iris bounding boxes) through a self-supervised learning mechanism. The more accurately the Decoder can synthesize rotated images, the more precisely the Finder can infer the corresponding torsional angle. Additionally, the rotation range adopted in this study for torsional nystagmus detection (−15° to +15°) is grounded in clinical reasoning. According to Kim et al. [24], a moderate torsional deviation of approximately 5° to 15° observed during pure or compound rotational nystagmus is considered pathologic, often associated with otoconial dislocation in the posterior semicircular canal rather than a benign physiological response. Furthermore, clinical neuro-ophthalmologic evaluations consider ocular torsional shifts within a 15° range to already constitute moderate or greater abnormalities [24]. Based on this evidence, the proposed system offers continuous estimation of torsional angles from VNG video sequences, providing clinicians with a quantitative measure to evaluate the severity of iris torsion and thereby enhancing the interpretability of rotational eye movements in clinical settings. In Figure 3, more detailed information of the torsion transformer can be seen.

As shown in Figure 3, the Decoder module of the Torsion Transformer receives the original image as input and processes it through (A) skip-connection blocks to generate feature maps used in (B) the anchor component. In (A), each input image of size 64 × 64 × 3 is independently passed through a skip-connection block, which consists of a 3 × 3 Conv2D layer. The convolutional layers sequentially extract 16 and then 64 features and finally reshape the feature map to 64 × 64 × 1. A ReLU activation function is applied, and the resulting feature map is concatenated with the original input image to yield a 64 × 64 × 2 tensor. This process preserves both the local features extracted via convolution and the spatial information of the original image. To exploit the strength of skip connections, the model extracts 16 and then 31 feature maps, which are finally reshaped to form the inputs for the anchor processing in (B). The skip-connection block operates on each of the three frames separately, resulting in three independent processing blocks. This architecture is motivated by the sensitivity requirement of the Finder module. The potential issue that training the Decoder on full sequences rather than single frames may increase the temporal gap between frames—thereby reducing torsional sensitivity per frame and possibly lowering estimation accuracy—has been further discussed in the Section 3.

The 31 feature maps generated through the skip-connection blocks are subsequently processed in (B) by anchor nodes, each of which corresponds to a specific angle. Instead of applying localized weight control, each anchor node performs a matrix multiplication across the entire feature map, allowing the model to emphasize global rotational features. The resulting representations are further processed within an “Anchor Block,” consisting of three Conv2D layers with 16, 64, and 1 filters, respectively. The final output is reshaped to 64 × 64 × 1 to facilitate comparison with the ground truth rotated image. The last step in anchor processing is a dropout operation: out of 31 anchor branches, only 3 are retained and the rest are discarded. Unlike conventional dropout, which operates at the neuron level, this dropout is performed at the block level to reduce redundancy. The final output from the Decoder is reshaped into a 64 × 64 × 3 tensor and supervised using a Mean Squared Error (MSE) loss function.

The final component, (C) the Finder, is designed to regress the torsional angle. The original input image is processed through a Conv2D layer, yielding a 64 × 64 × 31 tensor—this dimensionality reflects the use of the Decoder’s 31 anchor branches. These feature maps are then condensed into a 64 × 64 × 3 tensor via additional Conv2D layers and passed through a Conv2D layer with 12 filters, followed by a pooling layer that reduces the size to 32 × 32 × 12. The final Conv2D layer compresses this to 32 × 32 × 4, which is then activated using ReLU and forwarded to a fully connected (FC) layer. The FC network consists of two layers with 1024 and 31 neurons, respectively. The final FC output corresponds to one of the 31 angle classes used during training, and the target torsional angle is used as ground truth. To enhance robustness against outliers, the loss function for angle regression is based on the Huber loss, which improves upon the conventional categorical cross-entropy.

The outputs of the Torsion Transformer’s Decoder and Finder modules aim at two objectives: image reconstruction and angle regression, respectively. These objectives are mathematically formulated as Equations (1)–(3).

(1) $$L_{Decoder}=\frac{1}{N}\sum_{p=1}^{N}{\left(I_{GT}\left(p\right)-I_{Output}\left(p\right)\right)}^{2}$$

First, the Decoder loss shown in Equation (1) is designed to train the reconstructed rotated image $I_{Output}$ to closely match the ground truth image $I_{GT}$. Here, $I_{GT}$ represents the input image rotated by a specific ground truth angle $T_{GT}^{\left(i\right)}$. As depicted in Equation (1), $L_{Decoder}$ is defined as the mean squared error (MSE) between $I_{GT}$ and $I_{Output}$. In this equation, N represents the total number of pixels in the image, and p denotes the pixel index. Thus, the Decoder generates an image rotated by a specific angle $T_{GT}^{\left(i\right)}$, and as the output image becomes more similar to the ground truth, the loss $L_{Decoder}$ decreases. Next, the Finder loss described in Equation (2) is a regression loss designed to ensure that the predicted angle $T_{Pred}^{\left(i\right)}$ closely approximates the actual angle $T_{GT}^{\left(i\right)}$. This loss compares the actual torsion angle labels to the Finder outputs for each data point i. To enhance stability, we employed the Huber loss function, which calculates squared error within a range δ and switches to linear error beyond that range [25]. Here, the threshold δ is set to 1.0, M represents the total number of data points. The Huber loss is less sensitive to outliers by applying linear increments (weights) to larger rotation errors.

(2) $$L_{Finder}=\frac{1}{M}\sum_{i=1}^{M}\left\{\begin{array}{c} \frac{1}{2}{\left(T_{GT}^{\left(i\right)}-T_{Pred}^{\left(i\right)}\right)}^{2}\;\;\;\;\;\;\;\;\;\;\;\;\;\;if\left|T_{GT}^{\left(i\right)}-T_{Pred}^{\left(i\right)}\right|\leq \delta \;\;\;\;\;\;\\ \delta \cdot \left(\left|T_{GT}^{\left(i\right)}-T_{Pred}^{\left(i\right)}\right|-\frac{1}{2}\delta \right)\;\;else\;\;\;\;\;\;\;\;\;\;\;\;\;\;\;\;\;\;\;\;\;\;\;\;\;\;\;\;\;\;\;\;\; \end{array}\right.$$

(3) $$L_{TorsionTransformer}=L_{Decoder}+L_{Finder}$$

As indicated by Equation (3), the Torsion Transformer is trained using a supervised learning method, and its total loss is defined as the sum of the two losses (Equations (1) and (2)). By simultaneously optimizing both Decoder and Finder, a complementary effect occurs: the more accurately the Decoder learns to reconstruct rotated images, the more precisely the Finder predicts rotation angles.

### 2.4. Nystagmus Detection Model

**Figure 4:**
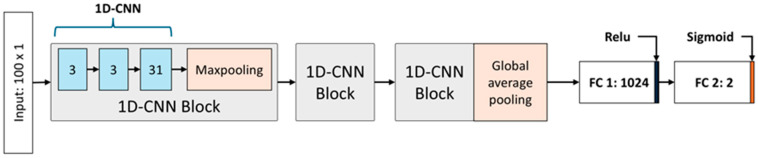
Architecture of the nystagmus detection model. The model takes a 100 × 1 sequence of torsional angles as input and is composed of three 1D-CNN blocks followed by two fully connected (FC) layers. Each 1D-CNN block consists of three 1D convolutional layers and one max-pooling layer. In the figure, gray boxes denote 1D-CNN blocks, blue boxes indicate 1D convolutional layers, and orange boxes represent pooling layers. The final block replaces max-pooling with global average pooling. The output passes through a fully connected layer with 1024 neurons, followed by a second FC layer with 2 neurons for binary classification.

Figure 4 illustrates the architecture of the nystagmus detection model designed to operate in conjunction with the proposed Torsion Transformer. This model takes as input the sequence of continuous torsional angles predicted by the Torsion Transformer and classifies whether nystagmus is present. The proposed nystagmus detection network adopts a shallow 1D-CNN-based architecture, chosen to minimize information loss and overfitting due to the low dimensionality and constrained range of the input data (−15° to +15°). This design choice is further supported by the work of Mun et al. (2024), which demonstrated strong performance in coordinate-based nystagmus detection using a structurally similar approach [16]. The model receives input in the form of a 100 × 1 torsional angle vector and is composed of nine 1D-CNN layers and two fully connected (FC) layers. The 1D-CNN layers are arranged into three identical groups of three layers each (3, 3, and 3), with each group forming a 1D-CNN block that includes one max-pooling layer. The overall structure comprises three stacked 1D-CNN blocks, where the final block replaces max-pooling with global average pooling. Max-pooling is applied to reduce feature map dimensionality while emphasizing salient features, whereas global average pooling is used in the final block to further compress the overall number of features. The two concluding FC layers serve as classifiers operating on the learned 1D-CNN feature maps. The final layer consists of two neurons and performs binary classification to distinguish between normal and nystagmus conditions.

The final feature map extracted through the three convolutional blocks is flattened and passed through two FC layers. A sigmoid activation function is applied at the output layer to compute a binary probability. Specifically, the output node represents a linear combination of inputs, and the application of the sigmoid function yields a value between 0 and 1, interpreted as the probability of nystagmus occurrence. In this study, a predicted value equal to or greater than 0.5 was classified as positive (i.e., presence of nystagmus). The detection model predicts the binary nystagmus label (0 = normal, 1 = nystagmus) based on the input time series of torsional angles. For each training sample, binary cross-entropy loss is computed between the predicted output and the ground truth label. To minimize this detection loss, the model parameters were optimized using the Adam optimizer. After training for more than 50 epochs, the model consistently achieved stable convergence. An early stopping criterion was employed: training was automatically terminated if the loss did not improve by more than 5% over three consecutive epochs compared to previous training performance.

## 3. Results

### 3.1. Performance Evaluation of Torsion Transformer for Torsion Prediction

**Figure 5:**
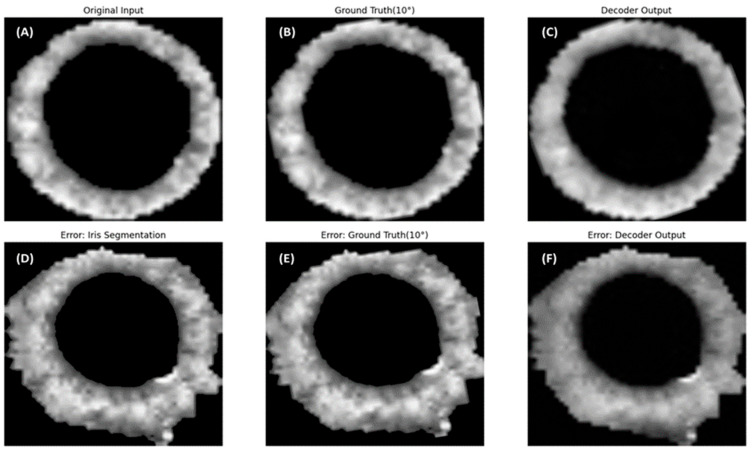
Representative cases of successful and failed torsional angle predictions by the Finder module. Panels (**A**–**C**) show examples in which the Finder accurately predicted the torsional angle. These images were also qualitatively evaluated as successful cases of iris segmentation. Specifically, (**A**) shows the original image, (**B**) is the same image rotated by 10 degrees, and (**C**) is the output generated by the Decoder using (**A**) as input. In contrast, panels (**D**–**F**) depict cases where the Finder failed to estimate the torsional angle accurately. These were qualitatively assessed as having segmentation errors during the iris extraction process. Panel (**D**) is the original image, which exhibits segmentation artifacts; (**E**) shows the image rotated by 10 degrees; and (**F**) is the Decoder-generated image based on (**D**) as input.

Table 1 summarizes the performance of the Decoder and Finder modules within the proposed Torsion Transformer. The Decoder module was trained to reconstruct rotated versions of input images, and its reconstruction accuracy was evaluated using the root mean squared error (RMSE) and the structural similarity index measure (SSIM). The evaluation dataset consisted of 42,165 images, including 29,514 normal images (70%) and 12,651 nystagmus-labeled images (30%). On the subset of images labeled as nystagmus, the Decoder achieved a difference of 0.97 in RMSE and 0.0112 in SSIM. Figure 5 illustrates example outputs generated by the Decoder alongside their corresponding original and ground truth rotated images. Notably, the image shown in Figure 5 was identified as a false prediction in the Finder module, despite having little impact on the Decoder evaluation metrics reported in Table 1.

**Figure 6:**
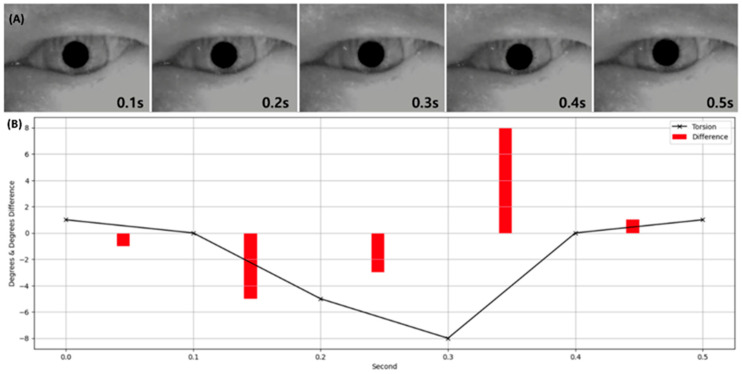
Visualization of the torsion output from the Finder module alongside the corresponding VNG image. Panel (**A**) displays a VNG image corresponding to a timepoint where torsional nystagmus was detected. Panel (**B**) presents a graph of the torsional angle predicted by the Finder, with values plotted at 0.1 s intervals. Red bars indicate the magnitude of change in torsion over time.

**Figure 7:**
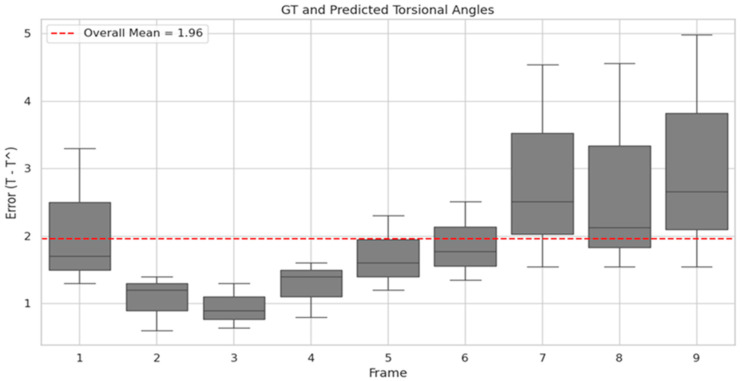
Distribution of torsional angle error across Transformer input frames. The x-axis represents the frame index of input images to the Transformer, while the y-axis shows the distribution of the difference between the predicted torsional angle (T^) by the Finder and the GT. The red line indicates the mean angular error across all frames.

The Torsion Finder classifies the torsional angle by leveraging the rotational features learned by the Decoder during image reconstruction, transmitted via shared anchor weights. The performance of the Finder was evaluated using classification accuracy, achieving an accuracy of 81.53%. The corresponding confusion matrix was as follows: True Positive (TP): 8994; False Positive (FP): 4131; True Negative (TN): 25,383; and False Negative (FN): 3657. Additional performance metrics beyond accuracy were as follows: sensitivity (recall) 0.7109, precision 0.6852, and specificity 0.8600. Figure 6 compares the torsional angles predicted by the Finder with the corresponding original VNG images, while Figure 7 visualizes the distribution of ground truth and predicted torsional angles across the Transformer’s input image frames.

### 3.2. Performance and Optimization of Nystagmus Detection Model

**Figure 8:**
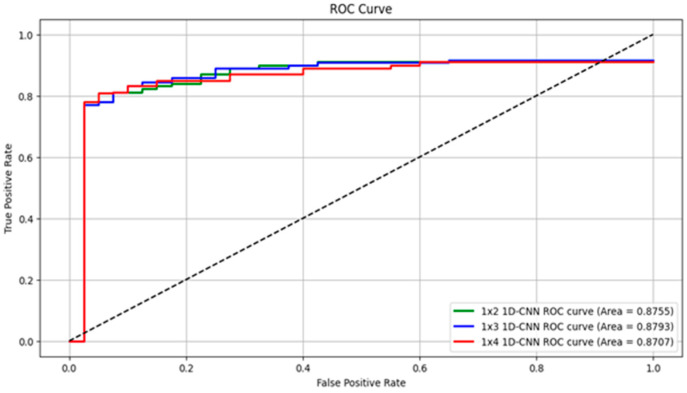
Presents ROC curves for the three best-performing models from Table 2. The green line represents the 0.2 s input model, the blue line indicates the 0.3 s model, and the red line corresponds to the 0.4 s model.

In this study, we proposed a 1D-CNN-based model to detect torsional nystagmus using torsional angles estimated by the Torsion Finder. Specifically, the Finder computes a single torsional angle from every sequence of three consecutive frames, yielding one torsion value per 0.1 s video segment. To optimize detection across varying durations of VNG segments, we evaluated model performance by altering the length of the sequential torsional angle input. Table 2 summarizes the performance metrics as the number of input torsion values increases from 2 to 9, corresponding to segment durations ranging from 0.2 to 0.9 s. The model using a 0.3 s VNG segment achieved the best overall performance, with 89.99% sensitivity, 86.36% specificity, 87.67% precision, 87.57% accuracy, and an F1-score of 88.82%. Notably, the model trained on 0.2 s segments achieved the highest specificity (88.37%) and precision (89.74%). However, performance gradually declined as the segment length exceeded 0.4 s, and dropped significantly below 80% after 0.6 s. Figure 8 presents the ROC curves of the top-performing models reported in Table 2. The model trained with 0.3 s segments achieved the highest area under the ROC curve (AUROC = 0.8793), followed by the 0.2 s model (AUROC = 0.8755) and the 0.4 s model (AUROC = 0.8707).

## 4. Discussion

This study proposed the Torsion Transformer to improve torsional nystagmus detection performance and support clinical decision-making. To evaluate the proposed Transformer and the accompanying 1D-CNN-based detection model, we utilized a dataset of 42,165 images, including 29,514 normal images (70%) and 12,651 images labeled as nystagmus (30%). As shown in Table 1, the Decoder achieved a reconstruction performance of RMSE = 27.895 and SSIM = 0.9264. The Finder module yielded an accuracy of 81.53%, with sensitivity (recall) of 0.7109, precision of 0.6852, and specificity of 0.8600. Notably, the Finder demonstrated lower sensitivity compared to accuracy, which we attribute to class imbalance (70% normal vs. 30% nystagmus) and error propagation from the Transformer. As shown in Figure 5, panels (D–F) represent input images that led to false predictions by the Finder. These images exhibited incorrect iris segmentation during preprocessing. The proposed Transformer framework mechanically generates both Decoder and Finder ground truths based on rotated versions of segmented iris images. Even when poor-quality segmentation images are input, the Decoder manages to generate outputs that closely resemble the rotated ground truth, as seen in Figure 5C,F. However, the Finder’s performance appears to degrade under such input conditions.

In designing the Torsion Transformer, emphasis was placed on optimizing Finder performance over the Decoder. As illustrated in Figure 7, the Finder’s prediction error relative to the ground truth torsional angle was evaluated across different input frame lengths. The average angular error from 1 to 9 input frames was 1.96°, with the lowest error observed at 3 frames. Although 2- and 4-frame inputs yielded comparable results, they showed greater variability than the 3-frame case. In particular, 1-frame inputs exhibited high variability—suggesting over-sensitivity—while 7- to 9-frame inputs showed under-responsive behavior. Given that one frame corresponds to approximately 0.03 s in VNG video, we infer that a 3-frame (0.1 s) segment is the optimal temporal resolution for the Finder module. Figure 6 visualizes the temporal variation in predicted torsional angles aligned with actual VNG sequences. In Figure 6A, the iris is clearly seen rotating counterclockwise between 0.1 and 0.3 s. In Figure 6B, the rotational velocity drops sharply between 0.2 and 0.3 s. At 0.4 s, a reversal to clockwise rotation is observed, which continues at a reduced speed for 0.5 s. Notably, the rapid clockwise rotation between 0.3 and 0.4 s is precisely captured, corresponding to a significant torsional change of approximately 8 degrees.

Our proposed framework carries important technical and clinical implications. Technically, we introduced a method for directly learning continuous rotational transformations. By restructuring detection as sequential angle regression followed by binary classification, the model more effectively captures the severity of nystagmus, enriching feature representations and enhancing performance. The multi-task learning between Decoder and Finder introduces a novel paradigm for better understanding of the data. Joint learning with image reconstruction enabled the model to intuitively internalize the concept of rotation, which substantially improved overall performance. As shown in Table 3, this approach achieved 89.99% sensitivity, 86.36% specificity, 87.67% precision, 87.57% accuracy, 88.82% F1 score, and 87.93% AUROC—on par with high-performing models for horizontal and vertical nystagmus detection.

Past studies on torsional nystagmus detection have largely relied on separate image processing and feature extraction techniques, or on adaptations of horizontal/vertical nystagmus detection methods. For instance, Zhang [17] proposed a method using pupil tracking and optical flow to capture torsional motion from patient videos, which were then classified by a Bi-stream CNN model. This approach required a complex preprocessing pipeline involving Hough transforms and template matching to correct pupil location, followed by optical flow generation that emphasized only torsional movement. The study reported a frame-wise F1-score of 81.0% and an accuracy of 85.73%, which were achieved after post-processing to correct label inconsistencies using temporal smoothing. In contrast, our study significantly reduces dependency on such preprocessing and post-processing by adopting an end-to-end deep learning framework. Apart from iris segmentation, the proposed Torsion Transformer learns rotational features directly, without optical flow calculations or manual feature engineering. Our detection model achieved a higher F1-score of approximately 88.8%, surpassing Zhang’s model in recall performance. Moreover, while Zhang’s method required post-processing of frame-wise predictions to identify nystagmus intervals, our method performs direct binary classification per 0.3 s window, making it more suitable for real-time continuous interpretation.

Lim [9] also proposed a deep learning model for 3D nystagmus recognition trained on extensive clinical datasets and reported improved performance over traditional methods. Their models often employed ResNet-based CNNs for frame-level feature extraction and temporal models like LSTM, or complex multi-stream architectures. Compared to these, our anchor-based Torsion Transformer quantitatively estimates precise torsional angles per frame, providing a more direct approach to capturing core nystagmus characteristics. Importantly, our adaptation of the Rotated Region Proposal Network (RPN) concept—originally used in general object detection—to estimate torsional eye rotation represents a notable innovation. Rotated RPN utilizes rotated anchors to predict the orientation of objects, overcoming limitations of traditional horizontal box detectors.

Li [6] proposed a BiLSTM model with the largest dataset in torsional nystagmus detection, achieving generalization with high sensitivity (91.20%), precision (94.30%), and accuracy (92.90%). However, this study lacked support for clinical decision-making, as it merely provided binary classification without offering interpretable information for clinicians. The most recent study by Krishna [26] employed a 2.5D-ResNet to detect torsional nystagmus, analyzing how image-based AI functions based on clinical context. While the model achieved 86.79% accuracy, 89.62% sensitivity, 83.96% specificity, and an AUC of 93.08, it similarly lacked the capacity to provide actionable insights for medical professionals.

Informed by the idea that AI models assessing torsional nystagmus often focus on the iris, we designed the Torsion Transformer to explicitly learn multiple angular features (anchors) by using rotational transformations of iris patterns. This mechanism operates analogously to comparing an input image against several latent image candidates rotated at various angles to determine the optimal match. Such an anchor-based approach simplifies learning, prevents local minima, and improves stability in torsion estimation compared to direct angle regression. Consequently, the proposed model, with its architecture specifically tailored for rotation analysis, achieved high accuracy even with relatively limited data. Most importantly, it provides interpretable, quantitative information for clinicians, thereby supporting direct clinical decision-making—a key distinction of this study.

**Figure 9:**
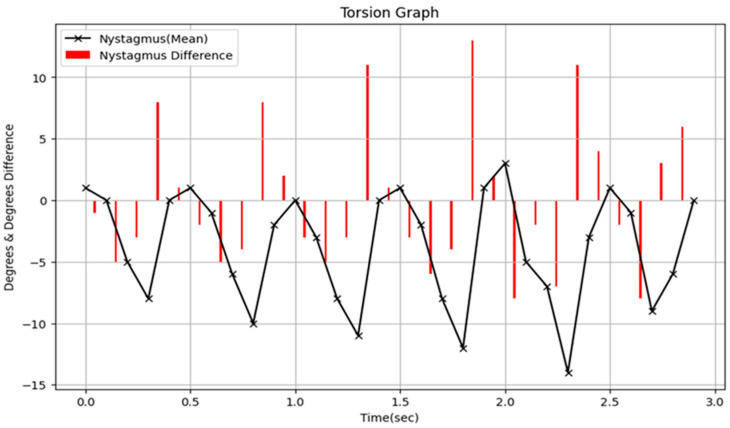
Presents a graph displaying torsional values over continuous time as derived from the VNG video. In this figure, the torsional angle is denoted by “x” and the corresponding variations in torsional angles are illustrated using red bars.

Clinically, this system provides valuable decision support that goes beyond simple binary outcomes (presence or absence of nystagmus). Unlike previous automated detection tools, our model outputs not only a binary classification of nystagmus but also quantitative measurements of torsional angles. This enables clinicians to objectively assess the severity of vestibular dysfunction and accurately track patient progress across multiple evaluations. Moreover, analyzing the time-series data of torsional angles can reveal temporal features such as onset latency, duration, and deceleration patterns, which may aid in differentiating specific variants of BPPV or identifying central-origin nystagmus. Figure 9 illustrates a graph of torsional values over continuous time output by the Torsion Transformer from a VNG video. As shown, this study provides decision-supportive information (torsional angle) rather than merely detecting the presence of torsional nystagmus. This allows for the quantification of periodicity, frequency, and duration of torsional nystagmus, thereby enabling clinical classification and analysis based on its type—previously discussed only qualitatively.

However, this study is not without limitations. First, it specifically focuses on torsional nystagmus in patients with BPPV. Although the detection of torsional nystagmus is clinically relevant, our findings may have limited generalizability to broader clinical contexts. This suggests the need for further validation across diverse vestibular disorders and clinical scenarios. Second, the proposed method heavily depends on accurate iris segmentation. In our approach, the segmented iris bounding box serves as a critical input to the Torsion Transformer, and segmentation errors can negatively affect the accuracy of torsional angle estimation. Addressing this limitation requires advancements in segmentation techniques and a deeper understanding of their interaction with downstream model components. As demonstrated in the failure examples shown in Figure 6, erroneous segmentation occasionally led to inaccurate torsion angle estimation. However, these inaccuracies did not consistently degrade the performance of nystagmus detection. The Torsion Finder achieved an accuracy of 81.53%, while the overall detection accuracy reached up to 87.70%. This implies that the intrinsic pattern recognition capabilities of the Transformer and detection models may mitigate the impact of segmentation errors on torsional nystagmus detection performance, though further experimental validation is warranted.

## 5. Conclusions

In conclusion, this study proposed a novel Torsion Transformer model for the automated detection of torsional nystagmus and validated its effectiveness using a clinical dataset. The model directly estimates ocular torsional angles from iris images and reliably classifies the presence of nystagmus using a 1D-CNN-based classifier. Experimental results demonstrated that the proposed framework achieves a detection accuracy comparable to state-of-the-art methods while also providing quantitative assessments of torsional angles. This contributes valuable support for clinical decision-making and offers insights into patient management.

Future research should address the limitations identified in this study, particularly regarding the Transformer modules, nystagmus detection model, and segmentation errors of the iris. Enhancing these components will further improve the performance of the proposed system. Additionally, integrating torsional detection with horizontal and vertical nystagmus detection approaches will broaden clinical applicability and promote standardization and efficiency in vestibular disorder diagnosis and management.

## Figures and Tables

**Table 1:** Decoder and Finder Performance of the Torsion Transformer.

Class = Subject(%)	RMSE ^1^ (STD)	SSIM ^2^	Accuracy
Normal = 29,514(0.7)	28.38 (±1.36)	0.9208	81.53
Nystagmus= 12,651(0.3)	27.41 (±1.21)	0.9320	

^1^ RMSE (Root Mean Square Error). ^2^ SSIM (Structural Similarity Index Measure).

**Table 2:** Optimized Performance of 1D-CNN-Based Nystagmus Detection Model.

Window Size	Input Time (s)	Recall	Specificity	Precision	ACC	F1 Score
2 × 1	0.2	87.13	88.37	89.74	87.70	88.42
3 × 1	0.3	89.99	86.36	87.67	87.57	88.82
4 × 1	0.4	88.97	85.46	87.61	87.34	88.28
5 × 1	0.5	82.81	82.01	84.59	82.44	83.69
6 × 1	0.6	78.30	77.20	79.56	77.79	78.93
7 × 1	0.7	78.00	76.58	78.80	77.33	78.40
8 × 1	0.8	78.15	75.77	78.26	77.02	78.20
9 × 1	0.9	77.93	76.58	78.79	77.29	78.36

**Table 3:** Presents a comparative summary of related prior studies.

Study (Year)	Target ^1^ (Quantitative Information) ^2^	Dataset No. Patients (No. VNG)	Method	Performance(%)					
				Recall	Specificity	Precision	ACC	F1 Score	AUROC
Ours (2025)	Torsional (Torsion)	127 (225)	1D-CNN Using Torsional Feature Components	89.99	86.36	87.67	87.57	88.82	87.93
Krishna et al. (2025) [26]	Torsional (No Measure)	72 (60)	2.5D ResNet	89.62	83.96	-	86.79	-	93.08
Li et al. (2023) [6]	Torsional (No Measure)	1236 (24,521)	BiLSTM	91.20	-	94.30	92.90	-	-
Zhang et al. (2021) [17]	Torsional (No Measure)	No reported (77)	TBSIN model with pupil position correction and optical flow	78.92	-	81.88	85.73	81.00	-
Lim et al. (2019) [9]	Torsional (No Measure)	No data reported for Torsion	2D-CNN using grid images	78.30	79.9	-	-	-	85.30

^1^ The “Target” column categorizes the type of nystagmus addressed into three distinct types. ^2^ Additionally, this column indicates whether the respective study incorporated quantitative metrics to support clinical decision-making.

## Data Availability

The data presented in this study are not publicly available due to ethical, legal, or privacy restrictions. However, the data may be made available upon separate review through the Data Safe Zone of Konyang University Hospital.
